# Identification of *Pseudomonas* Spp. That Increase Ornamental Crop Quality During Abiotic Stress

**DOI:** 10.3389/fpls.2019.01754

**Published:** 2020-01-28

**Authors:** Nathan P. Nordstedt, Laura J. Chapin, Christopher G. Taylor, Michelle L. Jones

**Affiliations:** ^1^ Department of Horticulture and Crop Science, Ohio Agricultural Research and Development Center, The Ohio State University, Wooster, OH, United States; ^2^ Department of Plant Pathology, Ohio Agricultural Research and Development Center, The Ohio State University, Wooster, OH, United States

**Keywords:** drought, floriculture, greenhouse production, high-throughput, horticulture, low nutrient, plant growth promoting rhizobacteria, plant-microbe interaction

## Abstract

The sustainability of ornamental crop production is of increasing concern to both producers and consumers. As resources become more limited, it is important for greenhouse growers to reduce production inputs such as water and chemical fertilizers, without sacrificing crop quality. Plant growth promoting rhizobacteria (PGPR) can stimulate plant growth under resource-limiting conditions by enhancing tolerance to abiotic stress and increasing nutrient availability, uptake, and assimilation. PGPR are beneficial bacteria that colonize the rhizosphere, the narrow zone of soil in the vicinity of the roots that is influenced by root exudates. In this study, *in vitro* experiments were utilized to screen a collection of 44 *Pseudomonas* strains for their ability to withstand osmotic stress. A high-throughput greenhouse experiment was then utilized to evaluate selected strains for their ability to stimulate plant growth under resource-limiting conditions when applied to ornamental crop production systems. The development of a high-throughput greenhouse trial identified two pseudomonads, *P. poae* 29G9 and *P. fluorescens* 90F12-2, that increased petunia flower number and plant biomass under drought and low-nutrient conditions. These two strains were validated in a production-scale experiment to evaluate the effects on growth promotion of three economically important crops: *Petunia* × *hybrida*, *Impatiens walleriana*, and *Viola* × *wittrockiana*. Plants treated with the two bacteria strains had greater shoot biomass than untreated control plants when grown under low-nutrient conditions and after recovery from drought stress. Bacteria treatment resulted in increased flower numbers in drought-stressed *P. hybrida* and *I. walleriana*. In addition, bacteria-treated plants grown under low-nutrient conditions had higher leaf nutrient content compared to the untreated plants. Collectively, these results show that the combination of *in vitro* and greenhouse experiments can efficiently identify beneficial *Pseudomonas* strains that increase the quality of ornamental crops grown under resource-limiting conditions.

## Introduction

Greenhouse-grown ornamentals are largely produced in containers using soilless growing mixes (Ball, 1998). Until recently, little attention has been given to the development of beneficial microbial communities within these containerized growing systems and to determining how these plant-bacteria associations can be used to improve ornamental crop quality. The rising costs of fertilizing and irrigating greenhouse crops have increased the interest in beneficial bacteria that can improve water and nutrient-use efficiency while reducing leaching and potential environmental contamination (Adesemoye et al., 2009). Plant growth promoting rhizobacteria (PGPR) can positively impact plant growth and resilience, resulting in a higher quality crop that is also more tolerant of drought and nutrient deficient conditions that might be encountered during shipping and retailing (i.e. postproduction abiotic stresses) (Waterland et al., 2010; Ruzzi and Aroca, 2015).

Plant growth promoting rhizobacteria (PGPR) comprise many taxonomic groups with diverse plant hosts (Kloepper et al., 1989). These bacteria colonize the rhizosphere, a narrow zone of soil that is associated with the roots and influenced by root exudates. In these beneficial plant-microbe interactions, the host plant secretes compounds into the rhizosphere that are used as a food source by the bacteria, which in turn stimulate plant growth and mediate stress responses through multiple mechanisms (Lugtenberg and Kamilova, 2009). PGPR can directly promote plant growth and abiotic stress tolerance by facilitating the acquisition of essential nutrients or by modulating the production of plant hormones (Goswami et al., 2016).

The application of bacteria that modify phytohormone concentrations such as gibberellins, abscisic acid, and auxin have been shown to increase osmotic stress tolerance and yield of soybean (Kang et al., 2014), reduce water loss in grape (Salomon et al., 2014), and increase rooting of kiwi (Erturk et al., 2010), respectively. Bacterial production of the enzyme ACC deaminase reduces production of the stress hormone ethylene in plants under drought (Glick, 2005), resulting in increased growth of tomato and pepper (Mayak et al., 2004), pea (Zahir et al., 2008), and maize (Zafar-ul-Hye et al., 2014) plants grown under water-limiting conditions. In addition, there is evidence that bacteria that withstand osmotic stress *in vitro* can confer this stress tolerance to plants (Asghar et al., 2015; Habib et al., 2016). The ability for bacteria to withstand osmotic stress is often attributed to their ability to form biofilms of exopolysaccharides (EPS), preventing desiccation under conditions of osmotic stress. Colonization of plant roots by EPS-producing bacteria increases tolerance to drought stress and increases shoot biomass of wheat (Hussain et al., 2014a) and root and shoot length of maize (Hussain et al., 2014b).

PGPR can improve plant nutrition by increasing nutrient availability, uptake, and assimilation. *Rhizobium* have been documented extensively for their ability to fix atmospheric nitrogen in symbiotic relationship with leguminous plants (Sessitsch et al., 2002). However, free-living nitrogen-fixing bacteria increase yield in corn (Garcia De Salamone et al., 1996; Kuan et al., 2016), plant size and nitrogen content of wheat (Sabry et al., 1997), and nitrogen uptake in tomato (Adesemoye et al., 2010). Phosphate solubilizing bacteria stimulate growth of many crops, resulting in increased yield and nutrient content of lettuce (Lai et al., 2008), improved germination and plant size of rice (Ashrafuzzaman et al., 2009), and increased berry production of raspberry (Orhan et al., 2006). PGPR that produce siderophores can chelate iron and make it more bioavailable to plants. Inoculation with these PGPR have been shown to increase iron content in plants (Zhou et al., 2018). The application of PGPR with the ability to enhance nutrient bioavailability have been used as a tool to reduce chemical fertilizer inputs without sacrificing crop quality (Adesemoye et al., 2009).

The genus *Pseudomonas* has been well studied for its ability to stimulate plant growth under drought and low-nutrient conditions (Jha and Saraf, 2015). Inoculation with *Pseudomonas* spp. results in an increase of root and shoot length and total plant biomass in sunflower, finger millet, and peas when subjected to drought conditions (Zahir et al., 2008; Sandhya et al., 2009; Chandra et al., 2018). Aspen seedlings grown under nutrient-limiting conditions and treated with *Pseudomonas fluorescens ﻿*strains Pf0-1, SBW25, and WH6 and *﻿P. protegens* Pf-5 have increased nutrient uptake and root length and biomass (Shinde et al., 2017). The *Pseudomonas putida* strain UW4 has served as a model system for studying the molecular and transcriptional properties of the enzyme ACC deaminase (Hontzeas et al., 2004; Cheng et al., 2008). The genus is also considered a model root colonizer, making it an optimal system to use in studying beneficial plant-microbe interactions (Lugtenberg et al., 2001).

Although there is growth in the area of PGPR research, much of this work has been conducted *in vitro* or focused on agronomic crops with little emphasis on ornamental crops (Paulitz and Richard, 2001; Vejan et al., 2016). There is evidence to suggest that PGPR tested *in vitro* often do not have the same growth-promoting effects when applied *in planta* (Ryu et al., 2005). In addition, many of these microorganisms originate from the soils of agronomic fields, and it is to be expected that changes in environment and abiotic factors would influence the efficacy of these organisms (Naylor and Coleman-Derr, 2018). Therefore, the inability to translate results from *in vitro* or field soil studies to greenhouse production of ornamental crops is of concern. The aim of this study was to identify *Pseudomonas* strains that stimulated growth and improved the quality of greenhouse-grown ornamentals under both drought and low-nutrient conditions.

## Materials and Methods

### Selection of Osmoadaptive Bacteria

An *in vitro* osmoadaptability bioassay was adapted from Asghar et al. (2015) for the selection of osmotic stress tolerant bacteria within a collection of 44 *Pseudomonas* strains originating from a variety of natural sources including water, soil, and plants (Mavrodi et al., 2012; Subedi et al., 2019). Briefly, single bacteria colonies were inoculated into separate wells of a 96-well microtiter plate pre-filled with 200 µL LB. Bacteria were incubated at 28°C with shaking at 120 rpm for 18 h. The optical density at 595 nm (OD_595_) was measured using a spectrophotometer (DTX880, Beckman Coulter, Brea, CA) and adjusted to OD_595_ of 0.8 with LB. Ten microliters of the bacteria cultures were transferred to a microtiter plate prefilled with tryptic soy broth (TSB) or yeast extract mannitol broth (YEM), each amended with 30% PEG_8000_ (w/v). Each bacteria strain was assayed on three separate microtiter plates for each media type (n = 3). Plates were incubated at 28°C with shaking at 120 rpm. After 96 h, the OD_595_ was measured to quantify the growth in PEG.

## *In Planta* Evaluation of Bacteria

### Experiment 1: High-Throughput Greenhouse Evaluation of Plant Responses to Treatment With Osmoadaptive Bacteria Strains

*Petunia × hybrida* ‘Picobella Blue’ seeds (Syngenta Flowers, Gilroy, CA) were sown in Pro-Mix PGX media (Premier Tech Horticulture, Quakertown, PA) and fertilized at each watering with 50 mg L^-1^ N from 15N–2.2P–12.5K–2.9Ca–1.2Mg water soluble fertilizer (JR Peters Inc., Allentown, PA) until transplant. Plants were grown ﻿in a greenhouse with temperatures set at 24/18°C (day/night) and a 16-h photoperiod. Supplemental lighting was supplied by high pressure sodium and metal halide lights (GLX/GLS e-systems GROW lights, PARSource, Petaluma, CA, USA) to maintain light levels above 250 μmol m^−2^ s^−1^. Seedlings were transplanted four weeks after sowing to 6.35 cm pots containing a 1:1 mix by volume of sand and turface (Profile Products LLC, Buffalo Grove, IL). Plants were arranged in a randomized complete block design (RCBD) with four blocks, and four single-plant replicates per block (n = 16).

To prepare bacteria inoculum, liquid LB media was inoculated with individual bacteria cultures selected from the *in vitro* osmoadaptability bioassay (**Table 1**) and incubated at 28°C for 9 h with shaking at 250 rpm. After incubation, cultures were adjusted to OD_595_ = 0.8 with LB. Final bacteria inoculum for treating the plants was prepared by diluting each culture 1:100 in reverse osmosis (RO) water. Uninoculated LB media diluted 1:100 with RO water was used as a negative control.

**Table 1:** *Pseudomonas* strains selected for their ability to withstand PEG-mediated osmotic stress *in vitro*.

Strain	Species	Source	Evaluated in multi-species greenhouse trial
14B11	*P. chlororaphis*	Missouri River	
14D6	*P. chlororaphis*	Mississippi River	
29G9	*P. poae*	Herbarium Sample	x
36B3	*P. fluorescens*	Wyoming Soil	
37D10	*P. brassicacearum*	Wyoming Soil	
48B8	*P. chlororaphis*	Wisconsin Soil	
48G9	*P. chlororaphis*	Wisconsin Soil	
89F1	*P. fluorescens*	Missouri Soil	
90F12-2	*P. fluorescens*	Missouri Soil	x
94G2	*P. frederiksbergensis*	Missouri Soil	

Selected strains were also evaluated *in planta* in a high-throughput greenhouse trial. Pseudomonas strains shown to increase plant growth in the high-throughput greenhouse trial were then evaluated in a multi-species greenhouse trial.

### Experiment 1a: Drought Stress

A greenhouse trial was developed to determine if bacteria application can enhance growth and recovery of petunia plants following drought stress. Following transplant, plants were fertilized at each irrigation with 50 mg L^-1^ N from 15N–2.2P–12.5K–2.9Ca–1.2Mg water soluble fertilizer (JR Peters Inc.). Plants were treated weekly with 40 mL bacteria inoculum or negative control LB solution, beginning the day after transplant. This volume saturated the growing media without resulting in leaching. Drought treatment began three weeks post-transplant by discontinuing weekly bacteria treatments and irrigation until all plants showed visible loss of turgidity across the plant. Plants were initially rewatered with RO water and regular irrigation with fertilizer and weekly bacteria treatments were then resumed. Plant performance was evaluated six weeks post-transplant. Flower numbers (including both open flowers and flower buds showing color) were counted and shoots (including stems, leaves, and flowers) were harvested. Tissue was dried in a forced-air oven at 49°C for at least 96 h and then weighed to measure shoot dry weight.

### Experiment 1b: Low-Nutrient Stress

A second greenhouse trial was developed to evaluate the effect of bacteria application on petunia plant growth under low-nutrient conditions. After transplanting, plants were maintained with 25 mg L^-1^ N from 15N–2.2P–12.5K–2.9Ca–1.2Mg water soluble fertilizer (JR Peters Inc.) at every irrigation to induce low-nutrient stress (Ball, 1998). Plants were also treated weekly with 40 mL bacteria inoculum as described previously. Uninoculated LB media was used as the negative control. Plant performance was evaluated as described for Experiment 1a.

### Experiment 2: Multi-Species Greenhouse Validation of Two *Pseudomonas* Strains

*Petunia × hybrida* ‘Picobella Blue’ (Syngenta Flowers), *Impatiens walleriana* ‘Super Elfin Ruby’ (PanAmerican Seed, West Chicago, IL), and *Viola × wittrockiana* ‘Delta Pure Red’ (Syngenta Flowers) seeds were sown and grown similar to Experiment 1. Seedlings were transplanted to 11.4 cm diameter pots containing Pro-Mix PGX (Premier Tech Horticulture) three weeks after sowing. Plants for each species were arranged in a RCBD with one plant per block. Due to variation in seed germination rates, there were 13, 14, and 18 blocks for *P. hybrida*, *V. wittrockiana*, and *I. walleriana*, respectively. Each species was blocked and analyzed independently. Bacteria inoculum was prepared according to the protocol in Experiment 1.

### Experiment 2a: Drought Stress

A greenhouse trial was developed to validate the effect of bacteria application on plant growth and performance after recovery from drought stress. The greenhouse trial was conducted similarly to Experiment 1a with modifications due to pot size. Each plant was treated weekly with 120 mL of bacteria inoculum or negative control LB solution to saturate the growing media without resulting in leaching. Drought treatment began five weeks post-transplant and plant performance was evaluated as described for Experiment 1 at nine weeks post-transplant. In addition, adhering media was washed from the roots, root tissue was dried in a forced-air oven at 49°C for at least 96 h, and root dry weight was used to calculate the root:shoot.

### Experiment 2b: Low-Nutrient Stress

A second greenhouse trial was developed to validate the effect of bacteria application on plant growth under low-nutrient conditions. The greenhouse trial was conducted similarly to Experiment 1b, and each plant was treated weekly with 120 mL of bacteria inoculum. Plant performance was determined by flower number and shoot biomass as described for Experiment 1, and root dry weight was determined as described in Experiment 2a to calculate the root:shoot. Plants were harvested at 8 weeks post-transplant. In addition, dried leaf and stem tissue was pooled for tissue nutrient analysis. At least three plants per treatment of each species were pooled per sample. Dried tissue was ground to pass through a 2-mm sieve. All nutrient analyses were conducted at the Service Testing and Research Laboratory (STAR Lab, The Ohio State University/OARDC, Wooster, OH). Total nitrogen analysis was conducted on a 100 mg sample using the Dumas combustion method (Vario Max combustion analyzer, Elementar America, Inc., Germany) (Sweeney, 1989). Following tissue digestion using a microwave system (Discover SP-D, CEM Corporation) and nitric acid digestion, a 250 mg sample was analyzed for P, K, Ca, Mg, and S using an inductively coupled plasma spectrometer (ICP)(model PS3000, Leeman Labs Inc., Hudson, NH) (Isaac and Johnson, 1985).

### Statistical Analysis

Statistical analyses were conducted in R Studio version 3.5.2 using an analysis of variance (ANOVA) with the model: Y = µ + treatment + block. Factors that had a significant p-value (p < 0.05) were analyzed using Tukey’s Honest Significant Difference.

## Results

### *In Vitro* Selection of Osmoadaptive Bacteria

**Figure 1:**
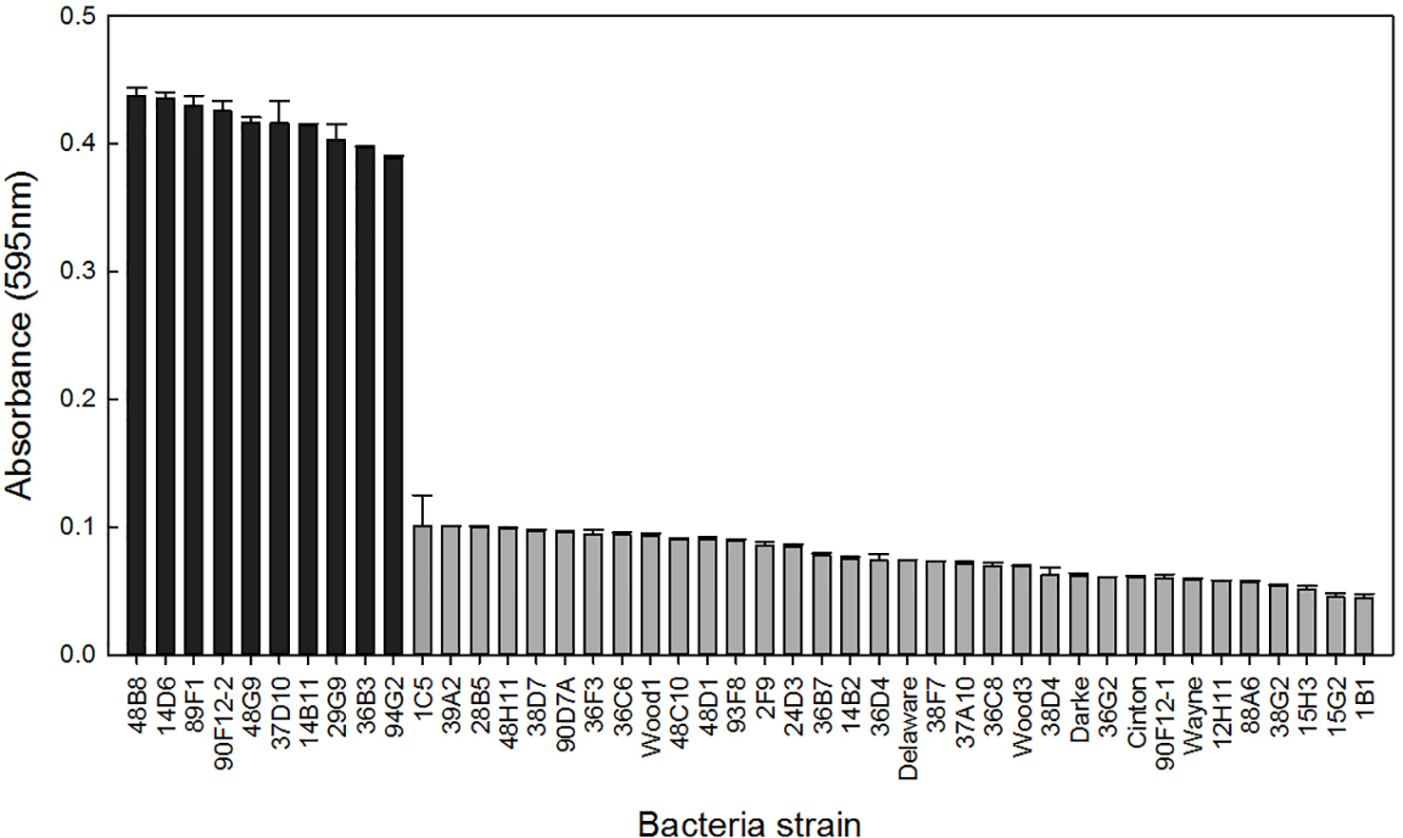
Bacteria strains were grown in YEM media containing 30% polyethylene glycol to induce osmotic stress. Bars represent the mean (± SE) optical density (595nm) of the strains after 96 h incubation at 28°C (n = 3). Strains with an absorbance greater than 0.3 (black) were selected for further evaluation.

A total of 44 *Pseudomonas* strains were screened for their ability to withstand osmotic stress *in vitro* by growing independently in Yeast Extract Mannitol (YEM) or Tryptic Soy Broth (TSB) media, both containing 30% polyethylene glycol (PEG). When grown in PEG-amended YEM, over 75% of the strains had an OD less than 0.1, and ten strains had at least 4-fold higher OD readings (**Figure 1**). These ten strains were selected for further evaluation based on their high level of osmoadaptability in the PEG-amended YEM (**Table 1**). No additional strains were selected in the TSB media containing PEG (data not shown).

### Development of a High-Throughput Greenhouse Trial to Evaluate the Efficacy of *In Vitro-*Selected Bacteria to Increase Plant Growth

**Figure 2:**
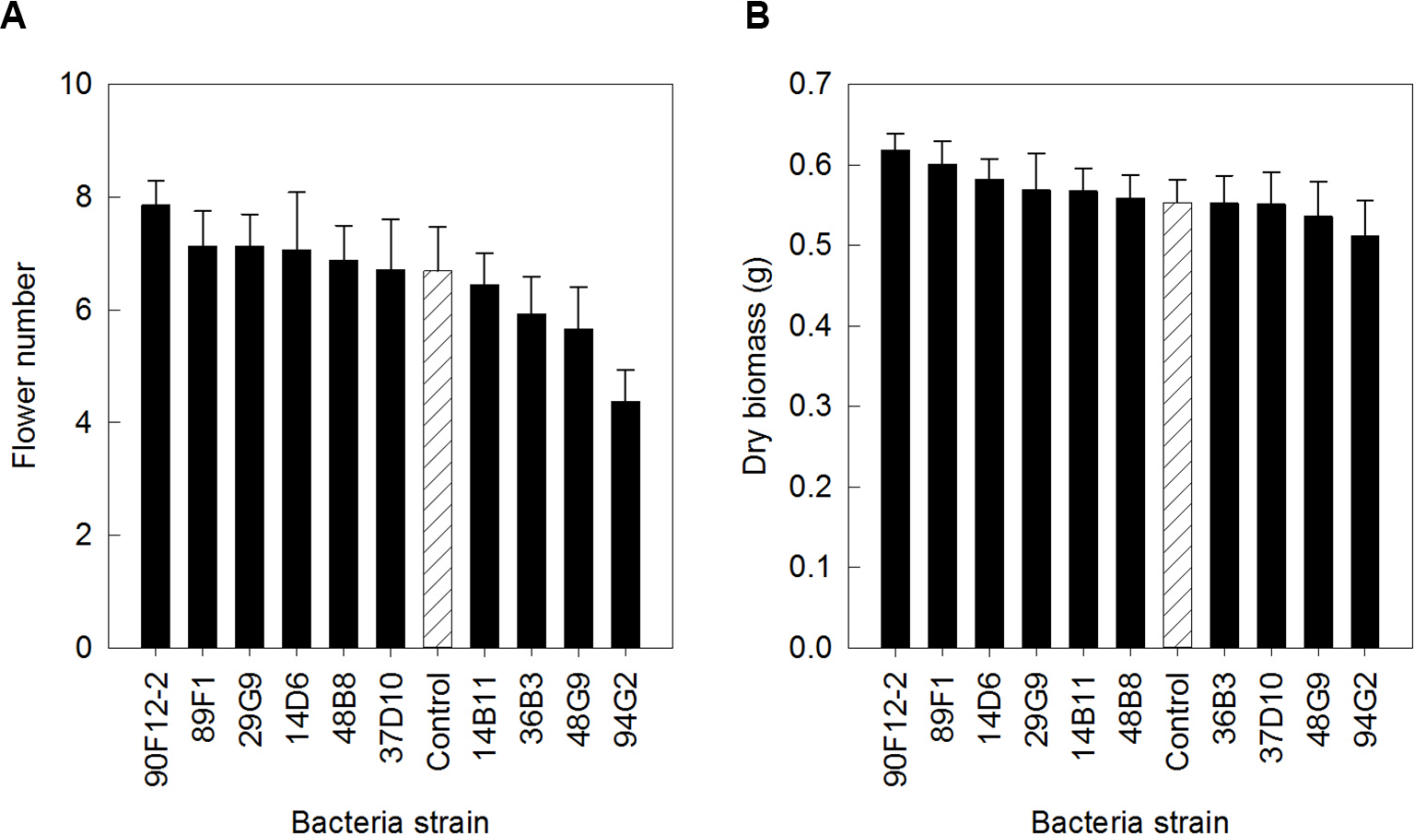
Plant growth performance parameters for *Petunia* ‘Picobella Blue’ plants subjected to drought stress three weeks after transplant (n = 16). Plants were treated with bacteria inoculum weekly after transplant (black) and compared to the uninoculated control (white with lines). Total number of flowers **(A)** and total shoot biomass (dry weight) **(B)** was measured two weeks after rewatering following drought stress. Due to the high-throughput nature of the trial, results were not statistically significant. Trends in plant growth promotion were used for selection. Bars represent mean (± SE).

**Figure 3:**
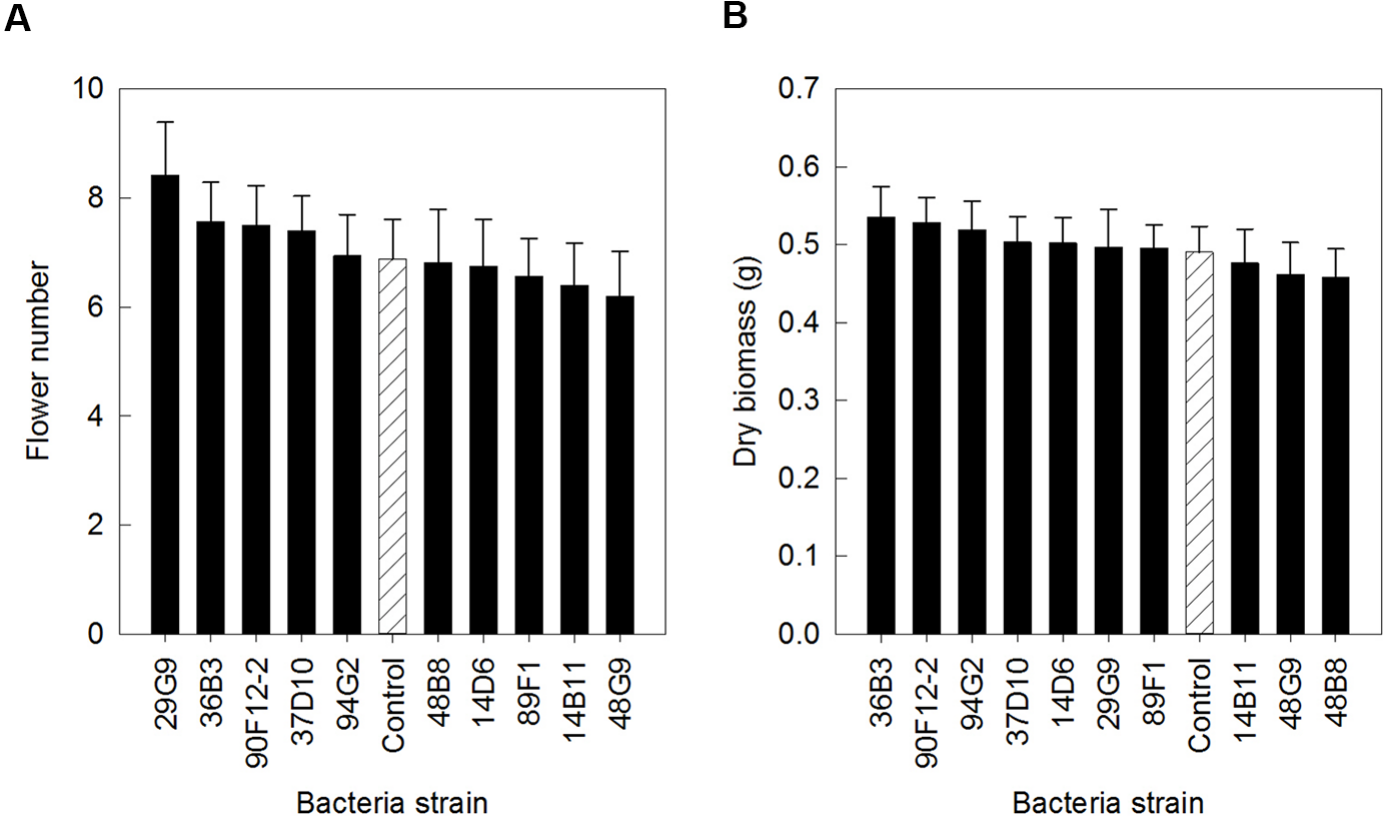
Plant growth performance parameters for *Petunia* ‘Picobella Blue’ plants grown with 25 mg L^-1^ N from 15N–2.2P–12.5K–2.9Ca–1.2Mg water soluble fertilizer at every irrigation to induce low-nutrient stress (n = 16). Plants were treated with bacteria inoculum weekly after transplant (black) and compared to the uninoculated control (white with lines). Total number of flowers **(A)** and total shoot biomass (dry weight) **(B)** was measured six weeks after transplant. Due to the high-throughput nature of the trial, results were not statistically significant. Trends in plant growth promotion were used for selection. Bars represent mean (± SE).

The ten *Pseudomonas* strains selected from the *in vitro* osmoadaptability bioassay were evaluated independently in drought and low-nutrient greenhouse trials for their ability to improve plant growth under abiotic stress as compared to the negative control. Although results were not statistically significant, there was a general increase in both shoot biomass and flower number in plants treated with bacteria. For petunia plants subjected to drought conditions, application of strains 90F12-2, 89F1, 29G9, 14D6, 48B8, and 37D10 increased the average flower number (**Figure 2A**) and strains 90F12-2, 89F1, 14D6, 29G9, 14B11, and 48B8 increased the average shoot biomass (**Figure 2B**), compared to untreated control plants. Of those strains, five bacteria increased both the average flower number and shoot biomass: 90F12-2, 29G9, 89F1, 14D6, and 48B8. Under low-nutrient conditions, application of strains 29G9, 36B3, 90F12-2, 37D10, and 94G2 increased the average flower number (**Figure 3A**) and strains 36B3, 90F12-2, 94G2, 37D10, 14D6, 29G9, and 89F1 increased the average shoot biomass (**Figure 3B**) of petunia plants. Of those strains, five strains increased the average of both flower number and shoot biomass: 29G9, 36B3, 90F12-2, 37D10, and 94G2. Due to the high-throughput nature of these trials, the trends in plant growth improvement were used to select for strains suitable for further evaluation. *Pseudomonas* strains 29G9 and 90F12-2 increased both average flower number and shoot biomass in petunias grown under both drought and low-nutrient conditions (**Figures 2** and **3**).

### Evaluation of Two *Pseudomonas* Strains to Increase Plant Growth and Stress Tolerance in Ornamental Crops

**Figure 4:**
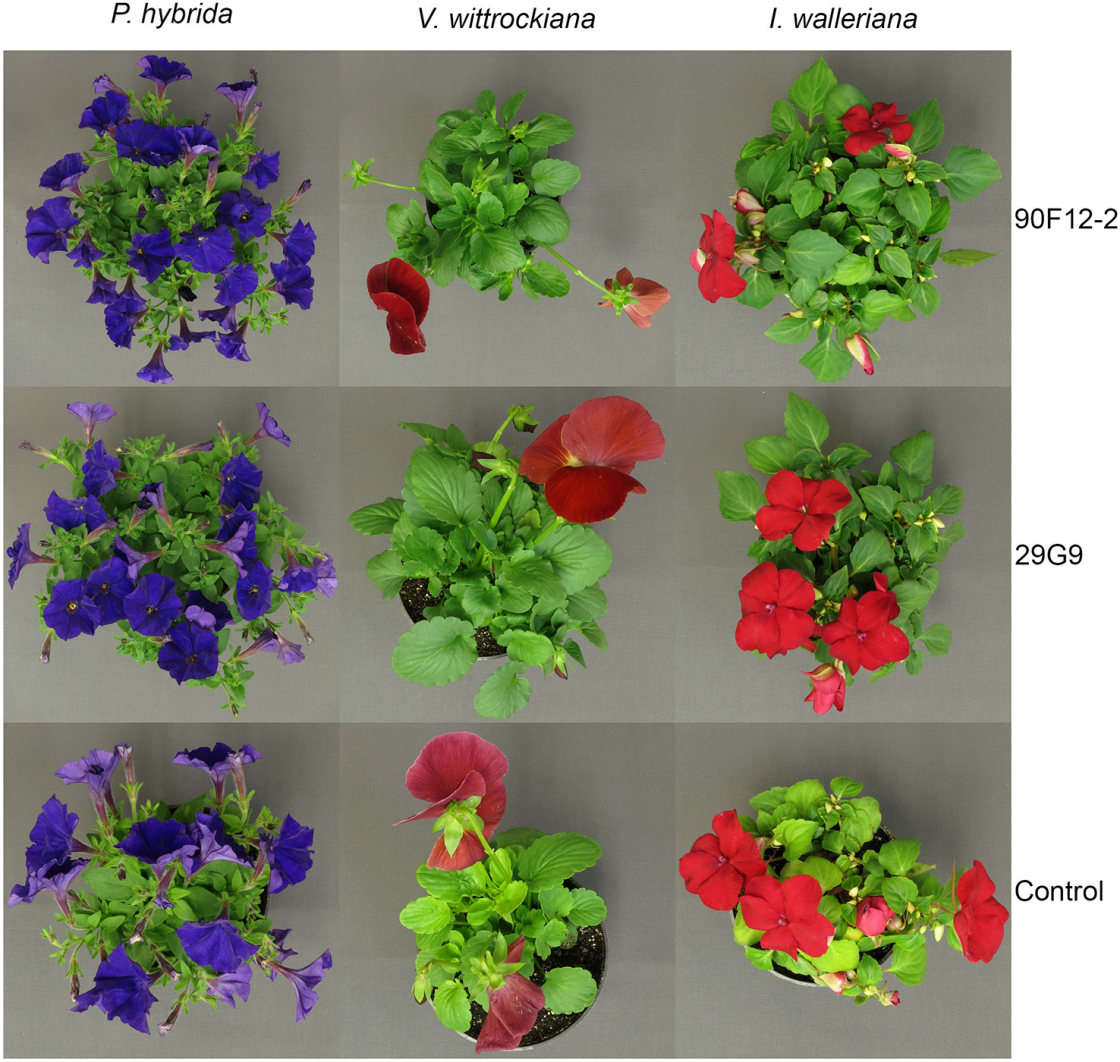
Visual crop quality of *Petunia* × *hybrida*, *Impatiens walleriana*, and *Viola* × *wittrockiana* plants eight weeks after transplant. Plants were grown with 25 mg L^-1^ N from 15N–2.2P–12.5K–2.9Ca–1.2Mg water soluble fertilizer at every irrigation to induce low-nutrient stress and treated weekly with *Pseudomonas* strains 90F12-2, 29G9, or uninoculated LB (control).

**Figure 5:**
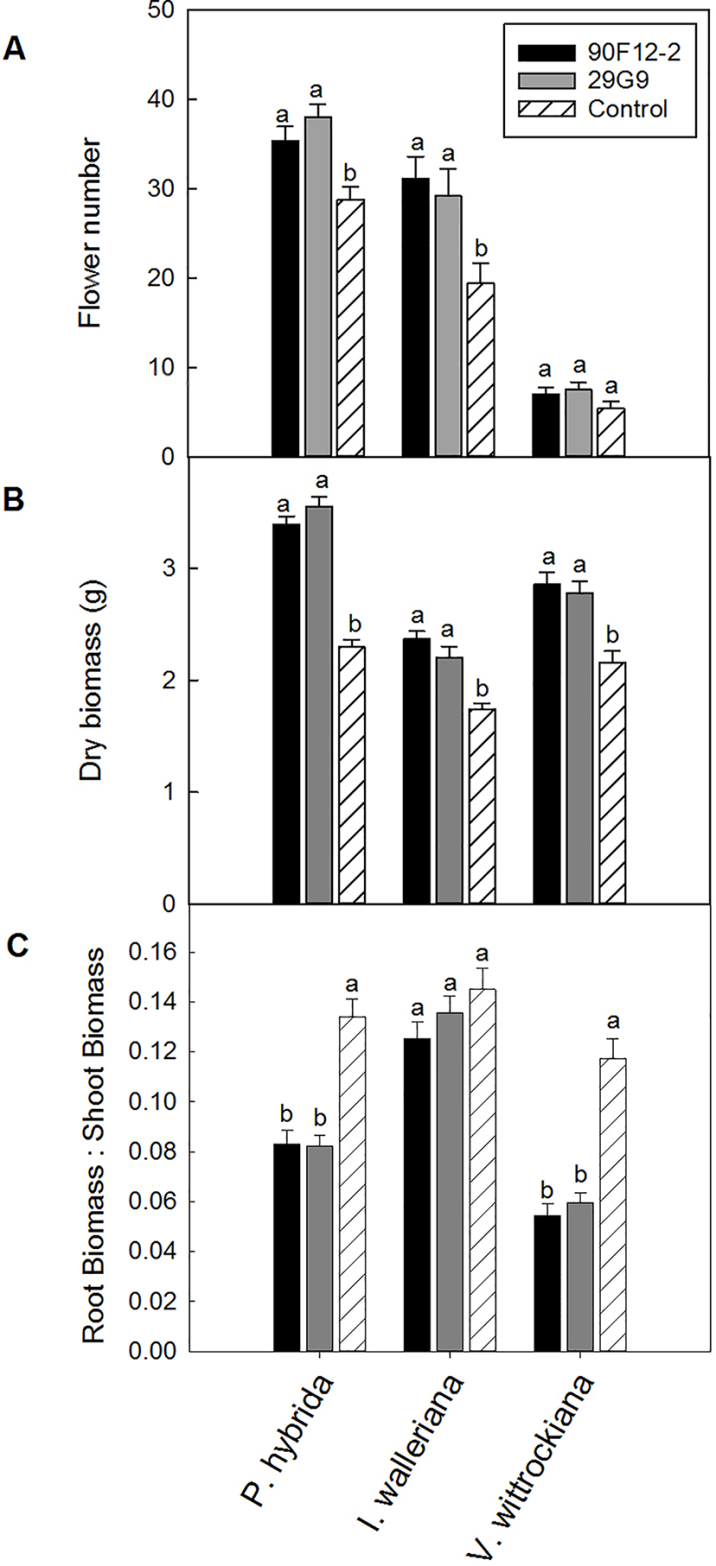
Plant growth performance parameters for *Petunia* × *hybrida*, *Impatiens walleriana*, and *Viola* × *wittrockiana* plants subjected to drought stress five weeks after transplant (n = 13, 18, 14). Plants were treated with bacteria inoculum weekly after transplant. Plants treated with strains 90F12-2 (black) and 29G9 (light gray) were compared to the uninoculated control (white with lines). Total number of flowers **(A)** and total shoot biomass (dry weight) **(B)** was measured two weeks after rewatering following drought stress. Root:shoot **(C)** was calculated with root and shoot dry weights. Bars represent the mean (± SE) with different letters representing significant difference (p < 0.05).

**Figure 6:**
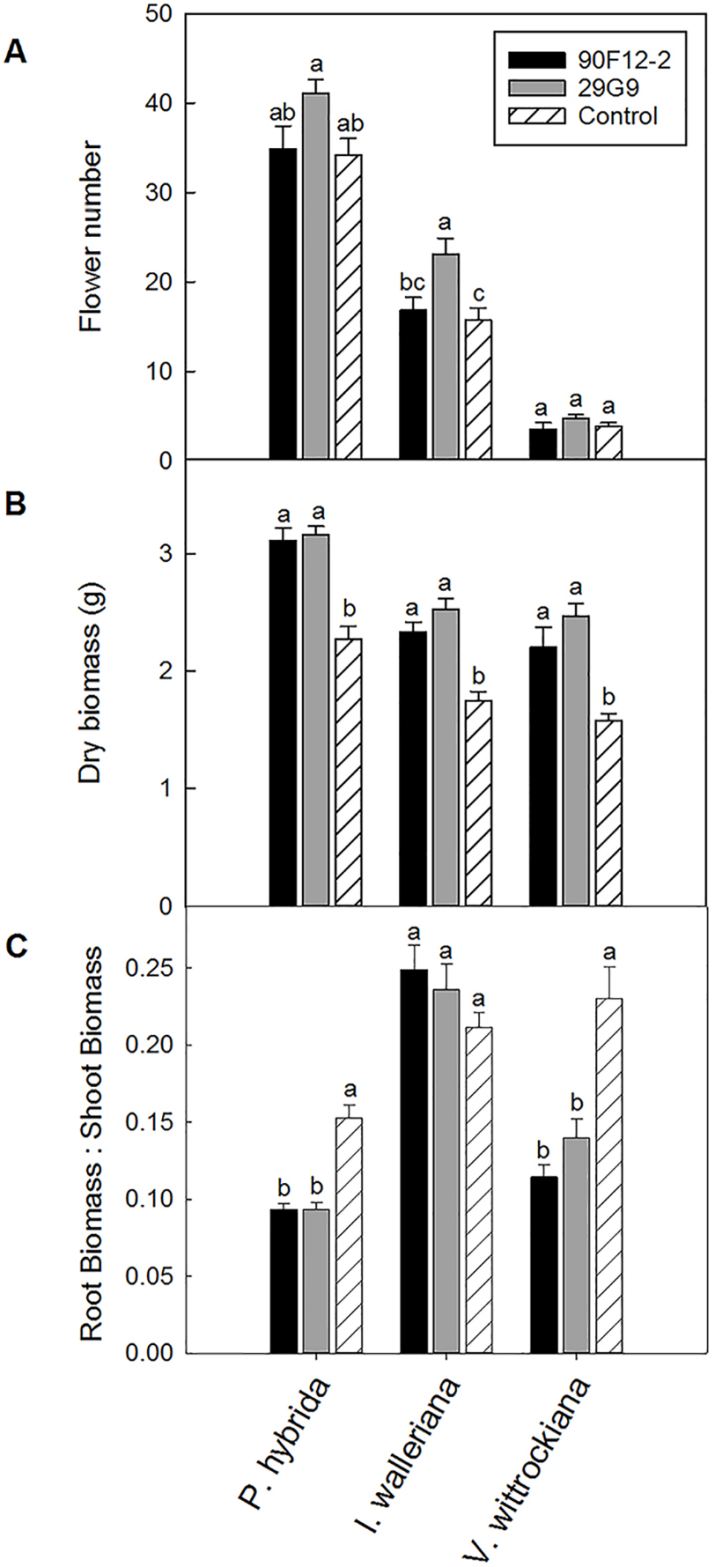
Plant growth performance parameters for *Petunia* × *hybrida*, *Impatiens walleriana*, and *Viola* × *wittrockiana* plants grown with 25 mg L^-1^ N from 15N–2.2P–12.5K–2.9Ca–1.2Mg water soluble fertilizer at every irrigation to induce low-nutrient stress (n = 13, 18, 14). Plants were treated with bacteria inoculum weekly after transplant. Plants treated with strains 90F12-2 (black) and 29G9 (light gray) were compared to the uninoculated control (white with lines). Total number of flowers **(A)** and total shoot biomass (dry weight) **(B)** was measured eight weeks after transplant. Root:shoot **(C)** was calculated with root and shoot dry weights. Bars represent the mean (± SE) with different letters representing significant difference (p < 0.05).

The two *Pseudomonas* strains identified in the high-throughput trial were evaluated for their ability to increase plant size and flower number in *P. hybrida*, *I. walleriana*, and *V. wittrockiana* subjected to drought and low-nutrient conditions. Overall, plants subjected to low-nutrient conditions and treated with each of the strains were visibly healthier, larger, and of higher quality compared to the uninoculated control plants. Control plants showed visible leaf yellowing, which was not observed in plants treated with bacteria (**Figure 4**). Application of the two bacteria strains significantly increased flower number and shoot biomass of *P. hybrida* subjected to drought stress by an average of 27% and 51%, respectively (**Figures 5A, B**). Under low nutrient conditions, application of each of the strains also increased *P. hybrida* shoot biomass by an average of 38% (**Figure 6B**). The root:shoot ratio of *P. hybrida* subjected to drought and low-nutrient stress was significantly lower for plants treated with each of the strains compared to the uninoculated control (**Figures 5C** and **6C**). For *I. walleriana* subjected to drought stress, the application of both strains increased flower number by an average of 55%, a 1.5-fold increase (**Figure 5A**). In addition, both strains increased shoot biomass of *I. walleriana* subjected to drought conditions by an average of 31% (**Figure 5B**). Strain 29G9 increased flower number by an average of 47% (**Figure 6A**), and both strains increased shoot biomass by an average of 39% (**Figure 6B**) for *I. walleriana* grown under low-nutrient conditions. There was no significant difference in root:shoot ratio of *I. walleriana* plants subjected to drought or low-nutrient conditions (**Figures 5C** and **6C**). In addition, there was no significant difference in flower number of *V. wittrockiana* plants treated with *Pseudomonas* and subjected to drought (**Figure 5A**) or low-nutrient conditions (**Figure 6A**) as compared to the uninoculated control. However, application of both strains increased shoot biomass of *V. wittrockiana* subjected to drought conditions by an average of 33% (**Figure 5B**) and increased the average shoot biomass of *V. wittrockiana* grown under low-nutrient conditions by 48%, a 1.5-fold increase (**Figure 6B**). Finally, there was a significant decrease in root:shoot ratio in *V. wittrockiana* plants subjected to drought and low-nutrient conditions and treated with both of the strains compared to the uninoculated control (**Figures 5C** and **6C**).

### Nutrient Content of Plant Leaf Tissue When Treated With Plant Growth-Promoting Rhizobacteria 

**Figure 7:**
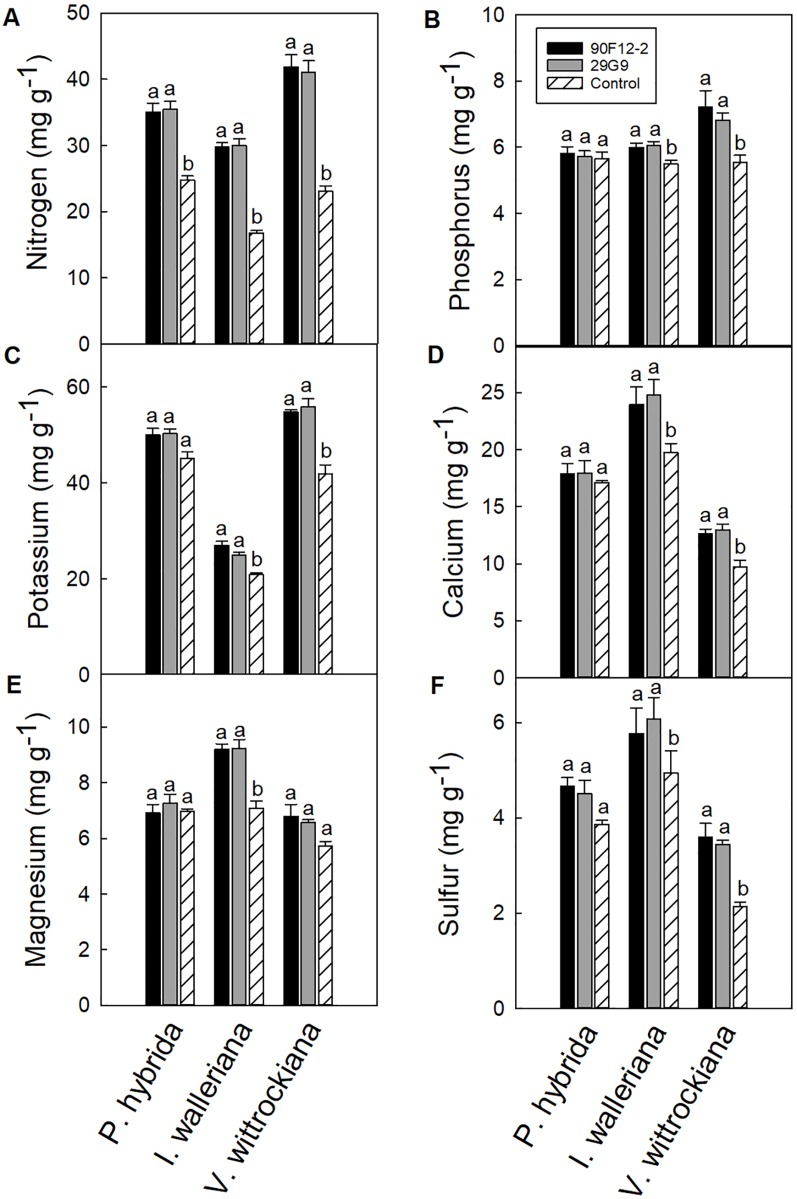
Leaf tissue nutrient content of *Petunia* × *hybrida*, *Impatiens walleriana*, and *Viola* × *wittrockiana* plants grown under low-nutrient conditions: nitrogen **(A)**, phosphorus **(B)**, potassium **(C)**, calcium **(D)**, magnesium **(E)**, and sulfur **(F)**. Plants were treated with bacteria inoculum weekly after transplant and tissue was harvested eight weeks after transplant. Plants treated with strains 90F12-2 (black) and 29G9 (light gray) were compared to the uninoculated control (white with lines). Bars represent the mean (± SE) with different letters representing significant difference (p < 0.05).

All plants were grown under low-nutrient regimes, which resulted in lower than optimum tissue nutrient content (Dole and Wilkins, 1999). On average, the application of bacteria resulted in higher shoot macronutrient content than plants that were not treated with bacteria (negative control). The application of each bacteria strain increased the nitrogen content of *P. hybrida* by at least 41% in plant leaf tissue compared to the uninoculated control (**Figure 7A**). There was no difference in the content of other nutrients in petunia leaf tissue. Similar to *P. hybrida*, both pseudomonads increased the nitrogen content of *I. walleriana* leaves by at least 78%. In addition, both strains increased the phosphorus, potassium, calcium, magnesium, and sulfur content of *I. walleriana* in plant leaf tissue by at least 9, 19, 21, 30, and 17%, respectively, compared to the negative control (**Figure 7**). The application of both strains increased the nitrogen content of *V. wittrockiana* leaf tissue by at least 78%. Both strains also increased phosphorus, potassium, calcium, and sulfur content of *V. wittrockiana* in plant leaf tissue by at least 23, 31, 30, and 62%, respectively, compared to the uninoculated control (**Figures 7A–D, F**). There was no difference in magnesium foliar nutrient content in *V. wittrockiana* leaf tissue between bacteria treatments and the negative control (**Figure 7E**).

## Discussion

Here, we established high-throughput *in vitro* and *in planta* assays to selected *Pseudomonas* strains that stimulate ornamental plant growth under water and nutrient deficient conditions. The use of PEG to select osmotic stress tolerant bacteria and the effect of these bacteria on plant tolerance to drought stress is well documented (Forchetti et al., 2007; Vardharajula et al., 2011; Hussain et al., 2014a; Asghar et al., 2015). Our study utilized a similar approach, validating the utility of this bioassay to screen larger collections of bacteria for their osmoadaptive properties. Similar to these previous studies, we found YEM growing media to be the most effective for selecting osmoadaptive bacteria. We utilized the PEG selection to narrow the collection of 44 Pseudomonads to ten osmotic stress tolerant strains (**Figure 1**). Our high-throughput greenhouse trial successfully confirmed that the bacteria could enhance plant stress tolerance and identified two strains that increased shoot biomass and flower number under both abiotic stress conditions (**Figures 2** and **3**). Studies have shown that application of osmotic stress tolerant bacteria originating from the rhizosphere and endosphere can stimulate plant growth under varied levels of drought stress. Bacteria able to grow *in vitro* in media supplemented with varying levels of PEG and subsequently evaluated *in planta* increase maize biomass and relative water content (Vardharajula et al., 2011), root length, and shoot biomass of wheat (Chakraborty et al., 2013; Asghar et al., 2015).

In addition to osmoadaptive properties, many PGPR exhibit other growth-promoting characteristics such as siderophore production, phosphate solubilization, and hormone regulation (Vardharajula et al., 2011; Chakraborty et al., 2013; Asghar et al., 2015). To further investigate the application of the *Pseudomonas* strains, we developed a multi-species greenhouse trial. Host specificity in plant-microbe interactions has been well-studied in both agronomic and horticulture crops, showing varied effects on plant growth depending on bacteria and plant genotype (Moutia et al., 2010; Pedraza et al., 2010). Due to species diversity in ornamental crop production systems, it is necessary to identify beneficial bacteria that can stimulate plant growth across a broad host range. Both *Pseudomonas* strains did not exhibit host specificity as they stimulated shoot growth of *P. hybrida, I. walleriana,* and *V. wittrockiana* under both drought and low-nutrient conditions. These results further demonstrate the broad utility of these bacteria in greenhouse production systems with a wide range of plant species that could encounter both drought and low-nutrient conditions. In addition, we found that the two *Pseudomonas* strains were able to colonize the rhizosphere of *P. hybrida* plants at least 8 weeks post-treatment (data not shown), supporting their ability to form a mutualistic relationship with the plants to stimulate plant growth. Our results support previous work that has shown general plant growth promotion of *Pelargonium peltatum, Chrysanthemum* sp., and *Dahlia variabilis* plants inoculated with *Pseudomonas* sp. (Göre and Altin, 2006).

Facilitating the uptake of nutrients by plants is one of the most common mechanisms that bacteria employ to stimulate plant growth. Some PGPR can fix atmospheric nitrogen, produce siderophores to provide iron to plant roots, solubilize mineral phosphate into a bioavailable form to plants, and synthesize different phytohormones and enzymes that can modulate plant development and lead to an increase in nutrient uptake (Glick, 1995). While positive growth responses were seen across all three plant species in our study, leaf nutrient content was more consistently increased in *I. walleriana* and *V. wittrockiana* plants treated with bacteria. Similarly, the visual symptoms of leaf yellowing in control plants compared to bacteria treated plants were more severe in *I. walleriana* and *V. wittrockiana* than *P. hybrida* (**Figure 4**). Previous studies evaluating the influence of bacteria inoculation on plants grown under low-nutrient conditions demonstrate a similar increase in foliar N and P content (Eid et al., 2009; Hoda and Mona, 2014), whereas strawberry plants treated with PGPR strains had an increase in N, P, K, Ca, Fe, Cu, and Mn foliar nutrient content (Ipek et al., 2014), and wheat plants grown under reduced fertility had an increase in N, P, K, Fe, Cu, Mn, and Zn when treated with different PGPR strains (Rana et al., 2012). Our study expands on this literature by documenting a similar plant response in a peat-based greenhouse media. Although our results varied depending on the bacteria treatment and plant species, increases in foliar N, P, K, Ca, Mg, and S content were observed (**Figure 7**). In addition, the significant decrease in the root:shoot ratio of inoculated *P. hybrida* and *V. wittrockiana* plants shows that plants treated with bacteria are able to sustain more shoot biomass with fewer roots compared to inoculated plants. This provides evidence that the bacteria are increasing bioavailability of nutrients or increasing nutrient use efficiency in plants, reducing the energy that plants have to expend to produce roots in search of adequate nutrients and likely contributing to the increase in overall crop quality (**Figure 4**).

Although many plant-PGPR studies are conducted in controlled environment greenhouses, these results can be difficult to translate to ornamental crop production due to the use of agronomic crop species and non-traditional soil-based growing media in these studies. PGPR strains have been evaluated as a tool to increase the quality of different ornamentals, demonstrating the ability of these bacteria to increase crop quality under varying conditions. As flower number is a major contributor to ornamental crop value, PGPR that increase flower number are of interest to the horticulture industry. *Bacillus subtilis* and *Glomus* sp. increase total flower number of *Tagetes erecta* plants when cultivated in a sandy loam soil (Flores et al., 2007), and inoculation with *Pseudomonas putida* and *P. fluorescens* strains increases plant size and flower number of *Pelargonium peltatum, Chrysanthemum* sp., and *Dahlia variabilis* plants cultivated under greenhouse conditions (Göre and Altin, 2006). In addition, application of the nitrogen-fixing *Azospirillum lipoferum* and the phosphate-solubilizing *Bacillus polymxa* increases the flower number of *Petunia* × *hybrida plants* grown under field conditions with different levels of fertility (Hoda and Mona, 2014). *Azotobacter chroococcum* and *Bacillus megaterium* have also been shown to increase plant growth and nutrient content of *Matthiola incana* plants when cultivated in soil under reduced and optimal fertility levels (Eid et al., 2009). Although our results varied across treatment, plant species, and stress, flower number was significantly increased in some scenarios. Application of both strains significantly increased flower number of *P. hybrida* subjected to drought stress, whereas application of strain 29G9 showed the most consistent positive effect across experiments, increasing flower number of *P. hybrida and I. walleriana* plants subjected to drought stress, and *I. walleriana* plants grown under low-nutrient conditions. No significant difference in flower number was observed between experiments with *V. wittrockiana*, which might be explained by the species’ habit of producing fewer flowers than other species. Our study expands on the current literature by evaluating the effect of bacterial application on economically-important ornamental species, grown in a peat-based growing media, and subjected to multiple abiotic stress conditions. Utilizing cultural inputs similar to those that would be used in commercial greenhouse facilities further validates the potential for these bacteria to be used across a wide range of growing conditions and abiotic stresses that can negatively impact crop quality.

*P. poae* strains increase plant growth of millet under drought conditions (Ranveer et al., 2016) and are capable of producing siderophores (Tian et al., 2009) and solubilizing phosphate (Woo et al., 2010; Ahmed et al., 2014), mechanisms typically associated with plant growth promotion. *P. fluorescens* strains also produce siderophores and solubilize phosphate (Goswami et al., 2016), in addition to stimulating growth of rice seedlings (Goswami et al., 2016) and acting as a biocontrol agent (Bhattacharyya and Jha, 2012).

This data will directly impact the scientific community studying beneficial plant-microbe interactions by providing efficient methods to translate *in vitro* selection to *in planta* observations. In addition, this information will benefit the horticulture industry by providing evidence that PGPR can be used as an effective tool to reduce fertilizer inputs without reducing crop quality. In conclusion, *in vitro* bioassays are effective at selecting candidate PGPR; however, their ability to stimulate plant growth should also be validated *in planta*. Our research has developed two greenhouse trials that can be used for the *in planta* validation of PGPR-mediated plant growth promotion under drought and nutrient-limiting conditions and in multiple plant species. Future research should focus on optimizing the application parameters such as application timing, concentration of bacteria inoculum, and incubation time of the bacteria culture.

## Data Availability Statement

The datasets generated for this study will not be made publicly available. All datasets generated for this study are included in the article/supplementary material.

## Author Contributions

NN, LC, and MJ conceived the experimental design. NN and LC collected the data. NN performed all analyses and led the writing of the manuscript. LC, CT, and MJ edited the manuscript. CT provided bacteria strains.

## Funding

Salaries and research support were provided in part by State and Federal funds appropriated to the OARDC, The Ohio State University. Journal Article Number HCS 19-12. This work was financially supported by the American Floral Endowment and the OSU D.C. Kiplinger Floriculture Endowment. Support was also provided by The Ohio State University Distinguished Fellowship and the OARDC Director’s Graduate Associateship.

## Conflict of Interest

The authors declare that the research was conducted in the absence of any commercial or financial relationships that could be construed as a potential conflict of interest.

## References

[B1] Adesemoye A. O. Torbert H. A. Kloepper J. W. (2009). Plant growth-promoting rhizobacteria allow reduced application rates of chemical fertilizers. Microb. Ecol. 58, 921–929. 10.1007/s00248-009-9531-y 19466478

[B2] Adesemoye A. O. Torbert H. A. Kloepper J. W. (2010). Increased plant uptake of nitrogen from 15N-depleted fertilizer using plant growth-promoting rhizobacteria. Appl. Soil Ecol. 46, 54–58. 10.1016/j.apsoil.2010.06.010

[B3] Ahmed E. A. Hassan E. A. Tobgy K. M. K. E. Ramadan E. M. (2014). Evaluation of rhizobacteria of some medicinal plants for plant growth promotion and biological control. Ann. Agric. Sci. 59, 273–280. 10.1016/j.aoas.2014.11.016

[B4] Asghar H. N. Zahir Z. A. Akram M. A. Ahmad H. T. Hussain M. B. (2015). Isolation and screening of beneficial bacteria to ameliorate drought stress in wheat. Soil Environ. 34, 100–110.

[B5] Ashrafuzzaman M. Hossen F. A. Razi Ismail M. Hoque A. Islam M. Z. Shahidullah S. M. (2009). Efficiency of plant growth promoting rhizobacteria (PGPR) for the enhancement of rice growth. Afr. J. Biotechnol. 8, 12471252.

[B6] Ball V. (1998). Ball redbook. 16th ed., Ed. Ball Batavia V. (IL: Ball Publishing).

[B7] Bhattacharyya P. N. Jha D. K. (2012). Plant growth-promoting rhizobacteria (PGPR): emergence in agriculture. World J. Microbiol. Biotechnol. 28, 1327–1350. 10.1007/s11274-011-0979-9 22805914

[B8] Chakraborty U. Chakraborty B. N. Chakraborty A. P. Dey P. L. (2013). Water stress amelioration and plant growth promotion in wheat plants by osmotic stress tolerant bacteria. World J. Microbiol. Biotechnol. 29, 789–803. 10.1007/s11274-012-1234-8 23239372

[B9] Chandra D. Srivastava R. Glick B. R. Sharma A. K. (2018). Drought-tolerant Pseudomonas spp. improve the growth performance of finger millet (Eleusine coracana (L.) Gaertn.) under non-stressed and drought-stressed conditions. Pedosphere 28, 227–240. 10.1016/S1002-0160(18)60013-X

[B10] Cheng Z. Duncker B. P. McConkey B. J. Glick B. R. (2008). Transcriptional regulation of ACC deaminase gene expression in Pseudomonas putida UW4. Can. J. Microbiol. 54, 128–136. 10.1139/W07-128 18388982

[B11] Dole J. M. Wilkins H. F. (1999). Floriculture priciples and species (Upper Saddle River, New Jersey: Prentice-Hall, Inc.).

[B12] Eid A. R. Awad M. N. Hamouda H. A. (2009). Evaluate effectiveness of bio and mineral fertilization on the growth parameters and marketable cut flowers of Matthiola incana L. Am. J. Agric. Environ. Sci. 5, 509–518.

[B13] Erturk Y. Ercisli S. Haznedar A. Cakmakci R. (2010). Effects of plant growth promoting rhizobacteria (PGPR) on rooting and root growth of kiwifruit. Biol. Res. 43, 91–98. 10.4067/S0716-97602010000100011 21157636

[B14] Flores A. C. Luna A. A. E. Portugal V. O. (2007). Yield and quality enhancement of marigold flowers by inoculation with Bacillus subtilis and Glomus fasciculatum. J. Sustain. Agric. 31, 21–31. 10.1300/J064v31n01

[B15] Forchetti G. Masciarelli O. Alemano S. Alvarez D. Abdala G. (2007). Endophytic bacteria in sunflower (Helianthus annuus L.): isolation, characterization, and production of jasmonates and abscisic acid in culture medium. Appl. Microbiol. Biotechnol. 76, 1145–1152. 10.1007/s00253-007-1077-7 17657487

[B19] Göre M. E. Altin N. (2006). Growth promoting of some ornamental plants by root treatment with specific fluorescent pseudomonads. J. Biol. Sci. 6, 610–615.

[B16] Garcia De Salamone I. E. Döbereiner J. Urquiaga S. Boddey R. M. (1996). Biological nitrogen fixation in Azospirillum strain-maize genotype associations as evaluated by the 15N isotope dilution technique. Biol. Fertil. Soils 23, 249–256. 10.1007/BF00335952

[B17] Glick B. R. (1995). The enhancement of plant growth by free-living bacteria. Can. J. Microbiol. 41, 109–117. 10.1139/m95-015

[B18] Glick B. R. (2005). Modulation of plant ethylene levels by the bacterial enzyme ACC deaminase. FEMS Microbiol. Lett. 251, 1–7. 10.1016/j.femsle.2005.07.030 16099604

[B20] Goswami D. Thakker J. N. Dhandhukia P. C. (2016). Portraying mechanics of plant growth promoting rhizobacteria (PGPR): A review. Cogent Food Agric. 2, 1–19. 10.1080/23311932.2015.1127500

[B21] Habib S. H. Kausar H. Saud H. M. Ismail M. R. Othman R. (2016). Molecular characterization of stress tolerant plant growth promoting rhizobacteria (PGPR) for growth enhancement of rice. Int. J. Agric. Biol. 18, 184–191. 10.17957/IJAB/15.0094

[B22] Hoda E. E.-M. Mona S. (2014). Effect of bio and chemical fertilizers on growth and flowering of Petunia hybrida plants. Am. J. Plant Physiol. 9, 68–77. 10.3923/ajpp.2014.68.77

[B23] Hontzeas N. Zoidakis J. Glick B. R. Abu-Omar M. M. (2004). Expression and characterization of 1-aminocyclopropane-1-carboxylate deaminase from the rhizobacterium Pseudomonas putida UW4: A key enzyme in bacterial plant growth promotion. Biochim. Biophys. Acta - Proteins Proteomics 1703, 11–19. 10.1016/j.bbapap.2004.09.015 15588698

[B24] Hussain M. B. Zahir Z. A. Asghar H. N. Asgher M. (2014a). Can catalase and exopolysaccharides producing rhizobia ameliorate drought stress in wheat? Int. J. Agric. Biol. 16, 3–13.

[B25] Hussain M. B. Zahir Z. A. Asghar H. N. Mahmood S. (2014b). Scrutinizing rhizobia to rescue maize growth under reduced water conditions. Soil Sci. Soc Am. J. 78, 538. 10.2136/sssaj2013.07.0315

[B26] Ipek M. Pirlak L. Esitken A. Figen Dönmez M. Turan M. Sahin F. (2014). Plant growth-promoting rhizobacteria (PGPR) increase yield, growth and nutrition of strawberry under high-calcareous soil conditions. J. Plant Nutr. 37, 990–1001. 10.1080/01904167.2014.881857

[B27] Isaac R. A. Johnson W. C. (1985). Elemental analysis of plant tissue by plasma emission spectroscopy: collaborative study. Assoc. Off. Anal. Chem. 68, 499–505.

[B28] Jha C. K. Saraf M. (2015). Plant growth promoting rhizobacteria (PGPR): a review. J. Agric. Res. Dev. 5, 108–119. 10.13140/RG.2.1.5171.2164

[B29] Kang S. M. Radhakrishnan R. Khan A. L. Kim M. J. Park J. M. Kim B. R. (2014). Gibberellin secreting rhizobacterium, Pseudomonas putida H-2-3 modulates the hormonal and stress physiology of soybean to improve the plant growth under saline and drought conditions. Plant Physiol. Biochem. 84, 115–124. 10.1016/j.plaphy.2014.09.001 25270162

[B30] Kloepper J. W. Lifshitz R. Zablotovicz R. M. (1989). Free-living bacteria inocula for enhancing crop productivity. Trends Biotechnol. 7, 39–44. 10.1016/0167-7799(89)90057-7

[B31] Kuan K. B. Othman R. Rahim K. A. Shamsuddin Z. H. (2016). Plant growth-promoting rhizobacteria inoculation to enhance vegetative growth, nitrogen fixation and nitrogen remobilisation of maize under greenhouse conditions. PloS One 11, 1–19. 10.1371/journal.pone.0152478 PMC4807084 27011317

[B32] Lai W. A. Rekha P. D. Arun A. B. Young C. C. (2008). Effect of mineral fertilizer, pig manure, and Azospirillum rugosum on growth and nutrient contents of Lactuca sativa L. Biol. Fertil. Soils 45, 155–164. 10.1007/s00374-008-0313-3

[B34] Lugtenberg B. Kamilova F. (2009). Plant-growth-promoting rhizobacteria. Annu. Rev. Microbiol. 63, 541–556. 10.1016/B978-0-12-374984-0.01169-4 19575558

[B33] Lugtenberg B. J. J. Dekkers L. Bloemberg G. V. (2001). Molecular determinants of rhizosphere colonization by Pseudomonas. Annu. Rev. Phytopathol. 39, 461–490. 10.1146/annurev.phyto.39.1.461 11701873

[B35] Mavrodi O. V. Walter N. Elateek S. Taylor C. G. Okubara P. A. (2012). Suppression of Rhizoctonia and Pythium root rot of wheat by new strains of Pseudomonas. Biol. Control 62, 93–102. 10.1016/j.biocontrol.2012.03.013

[B36] Mayak S. Tirosh T. Glick B. R. (2004). Plant growth-promoting bacteria that confer resistance to water stress in tomatoes and peppers. Plant Sci. 166, 525–530. 10.1016/j.plantsci.2003.10.025

[B37] Moutia J.-F. Y. Saumtally S. Spaepen S. Vanderleyden J. (2010). Plant growth promotion by Azospirillum sp. in sugarcane is influenced by genotype and drought stress. Plant Soil 337, 233–242. 10.1007/s11104-010-0519-7

[B38] Naylor D. Coleman-Derr D. (2018). Drought stress and root-associated bacterial communities. Front. Plant Sci. 8, 1–16. 10.3389/fpls.2017.02223 PMC5767233 29375600

[B39] Orhan E. Esitken A. Ercisli S. Turan M. Sahin F. (2006). Effects of plant growth promoting rhizobacteria (PGPR) on yield, growth and nutrient contents in organically growing raspberry. Sci. Hortic. (Amsterdam). 111, 38–43. 10.1016/j.scienta.2006.09.002

[B40] Paulitz T. C. Richard R. B. (2001). Biological control in greenhouse systems. Annu. Rev. Phytopathol. 39, 103–133. 10.1146/annurev.phyto.39.1.103 11701861

[B41] Pedraza R. O. Motok J. Salazar S. M. Ragout A. L. Mentel M. I. Tortora M. L. (2010). Growth-promotion of strawberry plants inoculated with Azospirillum brasilense. World J. Microbiol. Biotechnol. 26, 265–272. 10.1007/s11274-009-0169-1

[B42] Rana A. Saharan B. Nain L. Prasanna R. Shivay Y. S. (2012). Enhancing micronutrient uptake and yield of wheat through bacterial PGPR consortia. Soil Sci. Plant Nutr. 58, 573–582. 10.1080/00380768.2012.716750

[B43] Ranveer K. Yogendra S. G. Ishwar P. S. Suvigya S. Amit K. S. (2016). Impact of arbuscular mycorrhizal fungus, Glomus intraradices, Streptomyces and Pseudomonas spp. strain on finger millet (Eleusine coracana L.) cv Korchara under water deficit condition. Afr. J. Biotechnol. 14, 3219–3227. 10.5897/ajb2015.14479

[B44] Ruzzi M. Aroca R. (2015). Plant growth-promoting rhizobacteria act as biostimulants in horticulture. Sci. Hortic. (Amsterdam). 196, 124–134. 10.1016/j.scienta.2015.08.042

[B45] Ryu C.-M. Hu C.-H. Locy R. D. Kloepper J. W. (2005). Study of mechanisms for plant growth promotion elicited by rhizobacteria in Arabidopsis thaliana. Plant Soil 268, 285–292. 10.1007/s11104-004-0301-9

[B46] Sabry S. R. S. Saleh S. A. Batchelor C. A. Jones J. Jotham J. Webster G. (1997). Endophytic establishment of Azorhizobium caulinodans in wheat. Proc. R. Soc 264, 341–346.

[B47] Salomon M. V. Bottini R. de Souza Filho G. A. Cohen A. C. Moreno D. Gil M. (2014). Bacteria isolated from roots and rhizosphere of Vitis vinifera retard water losses, induce abscisic acid accumulation and synthesis of defense-related terpenes in in vitro cultured grapevine. Physiol. Plant 151, 359–374. 10.1111/ppl.12117 24118032

[B48] Sandhya V. Ali S. K. Z. Grover M. Reddy G. Venkateswarlu B. (2009). Alleviation of drought stress effects in sunflower seedlings by the exopolysaccharides producing Pseudomonas putida strain GAP-p45. Biol. Fertil. Soils 46, 17–26. 10.1007/s00374-009-0401-z

[B49] Sessitsch A. Howieson J. G. Perret X. Antoun H. Martínez-Romero E. (2002). Advances in rhizobium research. CRC. Crit. Rev. Plant Sci. 21, 323–378. 10.1080/0735-260291044278

[B50] Shinde S. Cumming J. R. Collart F. R. Noirot P. H. Larsen P. E. (2017). Pseudomonas fluorescens transportome ts linked to strain-specific plant growth promotion in aspen seedlings under nutrient stress. Front. Plant Sci. 8, 1–13. 10.3389/fpls.2017.00348 28377780 PMC5359307

[B51] Subedi N. Taylor C. G. Paul P. A. Miller S. A. (2019). Combining partial host resistance with bacterial biocontrol agents improves outcomes for tomatoes infected with Ralstonia pseudosolanacearum. Crop Prot. In press. 10.1016/j.cropro.2019.03.024

[B52] Sweeney R. (1989). Generic combustion method for determination of crude protein in feeds: collaborative study. Assoc. Off. Anal. Chem. 72, 770–774. 2808239

[B53] Tian F. Ding Y. Zhu H. Yao L. Du B. (2009). Genetic diversity of siderophore-producing bacteria of tobacco rhizosphere. Braz. J. Microbiol. 40, 276–284. 10.1590/s1517-83822009000200013 24031358 PMC3769708

[B54] Vardharajula S. Ali S. Z. Grover M. Reddy G. Bandi V. (2011). Drought-tolerant plant growth promoting Bacillus spp.: Effect on growth, osmolytes, and antioxidant status of maize under drought stress. J. Plant Interact. 6, 1–14. 10.1080/17429145.2010.535178

[B55] Vejan P. Abdullah R. Khadiran T. Ismail S. Nasrulhaq Boyce A. (2016). Role of plant growth promoting rhizobacteria in agricultural sustainability-a review. Molecules 21, 1–17. 10.3390/molecules21050573 PMC6273255 27136521

[B56] Waterland N. L. Campbell C. A. Finer J. J. Jones M. L. (2010). Abscisic acid application enhances drought stress tolerance in bedding plants. HortScience 45, 409–413. 10.21273/HORTSCI.45.3.409

[B57] Woo S.-M. Lee M. Hong I. Poonguzhali S. Sa T. (2010). Isolation and characterization of phosphate solubilizing bacteria from Chinese cabbage, in: 19th World Congress of Soil Science, Soil Solutions for a Changing World Conference Proceedings. pp. 56–59.

[B58] Zafar-ul-Hye M. Farooq H. M. Zahir Z. A. Hussain M. Hussain A. (2014). Application of ACC-deaminase containing rhizobacteria with fertilizer improves maize production under drought and salinity stress application of ACC-deaminase containing rhizobacteria with fertilizer improves maize production under drought and salinity St. Int. J. Agric. Biol. 16, 591–596.

[B59] Zahir Z. A. Munir A. Asghar H. N. Shaharoona B. Arshad M. (2008). Effectiveness of rhizobacteria containing ACC deaminase for growth promotion of peas (Pisum sativum) under drought conditions. J. Microbiol. Biotechnol. 18, 958–963. 18633298

[B60] Zhou C. Zhu L. Ma Z. Wang J. (2018). Improved iron acquisition of Astragalus sinicus under low iron-availability conditions by soil-borne bacteria Burkholderia cepacia. J. Plant Interact. 13, 9–20. 10.1080/17429145.2017.1407000

